# Management of postpartum haemorrhage

**DOI:** 10.12688/f1000research.7836.1

**Published:** 2016-06-27

**Authors:** Marie Pierre Bonnet, Dan Benhamou

**Affiliations:** 1 Department of Anaesthesia and Intensive Care Medicine, Paris Descartes University, Paris, France; 2 Department of Anaesthesia and Intensive Care Medicine, Paris Sud University, Paris, France

**Keywords:** coagulopathy, Postpartum Haemorrhage, transfusion strategy, tranexamic acid, fibrinogen

## Abstract

Postpartum Haemorrhage (PPH) is a major cause of maternal morbidity and mortality. Treatment of acquired coagulopathy observed in severe PPH is an important part of PPH management, but is mainly based on literature in trauma patients, and data thus should be interpreted with caution. This review describes recent advances in transfusion strategy and in the use of tranexamic acid and fibrinogen concentrates in women with PPH.

## Introduction

Postpartum hemorrhage (PPH) is one of the most frequent life-threatening complications of going into labor and occurs mostly without any warning or predictive signs or symptoms and often in the absence of predisposing conditions. The main causes of PPH are uterine atony, retained placenta, and genital tract trauma. Abnormal placentation, placental abruption, and uterine rupture are less frequent but often responsible for severe PPH with acquired coagulopathy. PPH accounts for nearly one-quarter of all maternal deaths worldwide and an estimated 125,000 deaths occur each year ^1^. Most of the time, these deaths due to obstetric hemorrhage are considered to be potentially preventable ^2, 3^. Maternal mortality is the end result of a worsening process, and PPH is also responsible for half of maternal morbidity ^4^. The incidence of PPH has recently increased in most developed countries such as Canada, Australia, and the US and has been notably related to an increased use of oxytocin for labor augmentation and subsequent uterine atony ^5^.

Currently, the therapeutic strategies for PPH management are largely standardized; in particular, obstetric, surgical, and radiological interventions play a life-saving role in PPH management. However, medical treatment, namely transfusion and a pro-hemostatic strategy, is also essential and has shown important changes in recent years. This review focuses on advances in transfusion strategy and on the use of pro-hemostatic agents such as tranexamic acid (TA) and fibrinogen concentrates in PPH.

## Transfusion strategy in postpartum hemorrhage

Only few data are available to guide transfusion management in the acute phase of PPH. The current guidelines are based mainly on the literature coming from trauma patients.

In trauma patients, several cohort studies have demonstrated a decrease in mortality associated with the administration of red blood cells (RBCs) and fresh frozen plasma (FFP) in a 1:1 ratio in the context of massive transfusion (10 units of RBCs or more) ^6– 9^. This strategy, which comes from a military setting and which is called damage control resuscitation, aims to administer coagulation factors as early as RBCs in order to treat blood loss but at the same time to prevent the coagulopathy observed in massively bleeding patients by limiting the use of crystalloids as volume replacement. These results are controversial. First, most of these studies were retrospective. Second, a survival bias cannot be ruled out ^10^. Indeed, the more severe trauma patients died early and were not able to be timely transfused in FFP, inducing an increased mortality in patients receiving a transfusion with a low FFP-to-RBC ratio. The transfusion benefit of a high FFP-to-RBC ratio is not so clear in recent prospective cohort studies. In the prospective, observational, multicenter, major trauma transfusion (PROMMTT) study documenting the timing of transfusion during active resuscitation in 905 trauma patients, Holcomb *et al*. demonstrated that early and higher FFP-to-RBC ratios were associated with a decreased mortality in patients transfused with at least three units of RBCs during the first 24 hours after admission, but not at 30 days ^11^. In a pragmatic randomized controlled trial from the same authors (the Pragmatic Randomized Optimal Platelet and Plasma Ratios [PROPPR] study), 680 severely injured patients requiring massive transfusion were randomly assigned between early administration of FFP, platelets, and RBCs in a 1:1:1 ratio compared with a 1:1:2 ratio. No significant difference in mortality at 24 hours or at 30 days was observed. However, in the 1:1:1 group, more patients achieved hemostasis and fewer experienced death due to exsanguination by 24 hours ^12^. Finally, the use of FFP is associated with an increased incidence of complications such as post-injury multiple organ failure, acute respiratory distress, and infections, and the rate of complications increased with the quantities of FFP transfused ^13^. For all of these reasons, the quality of the proofs in favor of a benefit in mortality with a transfusion in FFP and RBCs in a 1:1 ratio is considered low, and the optimal FFP-to-RBC ratio is still not determined. The recent European guidelines on coagulopathy management in trauma patients recommend an initial administration of plasma in patients with massive bleeding and, if further plasma is administered, an optimal FFP-to-RBC ratio of at least 1:2 is suggested. It is also recommended that FFP transfusion be avoided in trauma patients without substantial bleeding ^14^.

In the obstetrical setting, there is no study on the impact of the FFP-to-RBC ratio on maternal morbidity and mortality. As severe PPH may result in secondary coagulopathy similar to what is described in massive blood loss in patients with trauma injury, some experts have proposed extending the transfusion guidelines described in trauma patients to women with severe PPH ^15^. One study in obstetric hemorrhage evaluating this strategy of transfusion with a high FFP-to-RBC ratio was conducted by Alexander *et al*., who compared maternal outcomes between women who received whole blood only, women who received RBCs only, and women who received a combination of blood products ^16^. In this study, complications attributable to hypovolemia were significantly increased in the combination group as compared with the whole blood and RBC groups. However, an indication bias could not be excluded, as hemorrhage was more severe in the group of women who received the combination of blood products, with larger quantities of RBCs transfused and an increased incidence of hysterectomy. For the moment, by analogy with transfusion strategy in trauma patients, transfusion with a high FFP-to-RBC ratio (between 1:2 and 1:1) should be dedicated only for women with PPH requiring massive transfusion.

In collaboration with the local blood bank, some teams have successfully developed the concept of massive transfusion protocol, which includes a transfusion package, early and repeated monitoring of hemostatic competence, and an intervention algorithm which is modeled on existing protocols used in the trauma service ^17, 18^. In case of massive PPH, a package consisting of six RBC units, at least four FFP units, and one apheresis platelet unit is immediately released (without waiting for laboratory results); this treatment is repeated as long as bleeding is not controlled.

The only strong recommendation on blood transfusion in PPH is that women receive RBCs as soon as possible in case of massive PPH. Because cross-matched blood is not always available, maternity units should have immediate access (within 5 minutes) to O-negative blood. If the need is less pressing, group-specific blood can be made available more quickly than fully cross-matched blood. Consequently, all maternity units should have their own reserve of blood products if there is no blood bank on-site ^19^.

Finally, it appears that, more than the predetermined ratio, the early treatment of coagulopathy with FFP and platelets determines maternal morbidity and mortality.

Unfortunately, blood transfusion has its own adverse consequences. To decrease transfusion exposure and to control the bleeding, pro-hemostatic agents are used more and more often in women with PPH.

## Postpartum hemorrhage and tranexamic acid

TA is an antifibrinolytic agent that inhibits the activation of plasminogen into plasmin. Its use is now clearly established for the control and prophylaxis of menorrhagia ^20^. Its efficacy has also been proven in elective surgery such as orthopedic, vascular, hepatic, or urologic surgery and more recently in bleeding trauma patients ^21^.

A meta-analysis published in the *British Medical Journal* in 2012 pooled all of the randomized controlled trials comparing TA with no TA or placebo in surgical patients ^22^. The results showed that TA reduced the probability of receiving a blood transfusion by one-third in elective surgery, but its effects on thromboembolic events and mortality remained uncertain.

Concerning trauma patients, the CRASH-2 (Clinical Randomisation of an Antifibrinolytic in Significant Haemorrhage-2) multicenter trial showed that mortality was significantly decreased in trauma patients who received TA (1 g in 10 minutes, followed by a 1 g continuous infusion over the course of 8 hours) as compared with placebo. In particular, mortality due to hemorrhagic shock was decreased by 15% ^21^.

In the obstetrical setting, the literature focuses mostly on the prophylactic use of TA to prevent PPH, particularly in the context of elective cesarean delivery. The most recent meta-analysis exploring the preventive effect on PPH and safety of TA versus placebo or no treatment was published in 2015 by the Cochrane database ^23^. It included nine trials involving 2453 women who were at low risk of PPH and who were undergoing cesarean delivery and three trials with 832 women who delivered vaginally. These trials were of mixed quality. The results of the meta-analysis showed that overall the incidence of blood loss of greater than 500 mL was lower in women who received TA versus placebo or no intervention. Also, TA was effective in decreasing the incidence of blood loss of greater than 1000 mL in women who had undergone cesarean delivery but not vaginal birth. Mean blood loss within the first 2 hours postpartum was lower in women who received TA, and this effect was similar following vaginal and cesarean birth, but the mean difference in blood loss reduction was very moderate; the mean difference was 78 mL (95% confidence interval of 58 to 98 mL). Blood transfusion was less frequent in women receiving TA versus placebo or no intervention. Finally, the authors found that the use of TA was associated with only mild side effects, such as nausea, vomiting, and dizziness.

Despite these encouraging results, the routine use of TA as a preventive treatment of PPH is currently not recommended and this was because of the moderate quality of the available evidence, the limited benefit on blood loss, and the lack of data on the thromboembolic risk.

Concerning the use of TA as a curative treatment of PPH, only one randomized trial has been published until now. In this open-labeled controlled study, 144 women with a blood loss of 800 mL or more after vaginal delivery were randomly assigned to receive either 4 g of TA within 1 hour followed by 1 g per hour during 6 hours or not ^24^. In the treatment group, total blood loss at 6 hours after PPH diagnosis was significantly lower, but the difference was questionable (170 versus 221 mL; *P* = 0.041). Duration of hemorrhage was also significantly shorter in the treatment group, and transfusion requirement and incidence of severe hemorrhage were lower.

To summarize, data on the effectiveness of TA in PPH are quite encouraging, but there is still only little reliable evidence coming from randomized controlled trials. However, owing to its low cost and low rate of side effects, the use of TA is currently recommended by several academic societies. For example, the most recently updated PPH treatment guidelines prepared by the World Health Organization state that TA (1 g over 5 minutes, repeated within 30 to 60 minutes if necessary) is recommended for the treatment of PPH if oxytocin and other uterotonics fail to stop bleeding or if it is thought that the bleeding may be partly due to trauma (weak recommendation) ^25^. Consequently, there is an urgent need for clinical randomized trials of good quality before TA can be strongly recommended as a curative treatment in PPH.

The World Maternal Antifibrinolytic (WOMAN) trial conducted worldwide aims to determine the effect of the early administration of TA on mortality and hysterectomy rates as well as major complication rates in women with clinically diagnosed hemorrhage ^26^. Inclusions are over now and the results of this study are due to be published later this year.

## Fibrinogen concentrates in postpartum hemorrhage

Fibrinogen plays a critical role in achieving and maintaining hemostasis and is fundamental to effective clot formation. In the context of massive obstetric bleeding, fibrinogen is the first coagulation factor to decrease ^27^; rapid fibrinolysis has also been described in some specific causes of PPH, such as placental abruption, placenta previa, genital tract trauma, and uterine atony.

Fibrinogen plasma level has been demonstrated to be a good predictor of PPH severity ^28, 29^. In the study by Charbit *et al*., a fibrinogen plasma level of 2 g/L or less had a 100% positive predictive value for severe PPH ^28^. This study also demonstrated that the risk for severe PPH was 2.6 fold higher for each 1 g/L decrease in fibrinogen plasma level. Therefore, the assumption that fibrinogen supplementation could be beneficial to treat PPH has been made, although this is likely an over-interpretation of the study results. It should indeed be noted that the study by Charbit *et al*. was not randomized and did not demonstrate that decreased fibrinogen concentration was a causal factor of PPH severity. This study demonstrated only that decreased fibrinogen concentration was associated with PPH severity. Therefore, basing our hemostatic strategy on this argument requires further study.

FFP transfusion is not the optimal agent for treating fibrinogen deficiency, as a volume of 30 mL/kg is necessary to increase the fibrinogen concentration by 1 g/L, inducing a high risk of fluid overload. In the past, fibrinogen therapy was usually given as cryoprecipitate, but owing to the potential viral contamination and variable concentration of fibrinogen in cryoprecipitate, human plasma-derived fibrinogen concentrates are now available in most countries but not everywhere. For example, in the UK, the only licensed source of fibrinogen is FFP or cryoprecipitate, which also contains von Willebrand factor, factor VIII, factor XIII, and fibronectin. Fibrinogen concentrates offer rapid restoration of the fibrinogen concentration with a small-volume infusion, for a comparable cost, and with a minimal preparation time. Fibrinogen concentrates are considered by many to be preferable to cryoprecipitate, although there are no studies comparing the efficacy of these two products ^30^.

The efficacy and safety of fibrinogen concentrates have been proven in congenital fibrinogen deficiencies ^31^. Additionally, some *in vitro* and animal studies with thromboelastography monitoring have shown that the addition of fibrinogen concentrates corrects the coagulation disorders induced by experimental hemodilution ^32, 33^. Clinical data on the efficacy of fibrinogen concentrates in the management of hemorrhage are still scarce. Overall, fewer than 10 randomized controlled trials have explored the potential benefit of fibrinogen concentrates in terms of transfusion requirement and correction of hemostasis disorders analyzed either by traditional hemostatic monitoring or by point-of-care (POC) viscoelastic methods ^34– 41^. These trials included 384 patients overall and were all performed in the setting of perioperative bleeding in scheduled cardiovascular surgery, except one trial including patients undergoing radical cystectomy ^35^. Overall, five trials found a decrease in transfusion requirement in patients who received fibrinogen concentrates ^34, 35, 39– 41^, and five trials described an increased clot firmness measured by thromboelastometry ^35, 38– 41^. However, only one study found a significant reduction in blood loss, which was not associated with a transfusion-sparing effect ^37^, and none of these trials found a significant difference in mortality rate. These trials have several methodological flaws: small sample size, no prolonged follow-up, no intention-to-treat analysis, and no or poor blindness design. The protocol of fibrinogen concentrate administration differed between studies in terms of dose and of therapeutic target (prophylactic or curative treatment) as well as the control group. Consequently, it is difficult to extend these results to the obstetrical setting.

In trauma patients, only observational studies have been published ^42^. In retrospective studies, the administration of fibrinogen concentrates was associated with a decrease in transfusion needs and with the correction of biologic hemostatic disorders ^43– 46^. In one of these studies, the observed mortality was lower than predicted mortality for patients who received fibrinogen concentrates ^47^. However, these retrospective studies also have several methodological flaws. In particular, the severity of hemorrhage and confounding factors concerning blood loss volume were not taken into account, inducing an indication bias. No randomized controlled trial on the impact of fibrinogen concentrates in trauma patients has yet been published. To summarize, data on fibrinogen concentrate efficacy and safety in bleeding trauma patients are too limited for a conclusion to be drawn.

The use of fibrinogen concentrate in PPH has been explored in seven observational studies, in which a total of 222 women participated ^43, 48– 51^. In six studies, a significant increase in fibrinogen plasma level was described after the administration of fibrinogen concentrate, but without a control group, the efficacy of fibrinogen concentrates cannot be determined. The only controlled study is a before-and-after study of 77 women with PPH ^52^. In this retrospective study, maternal outcomes were compared between women who received cryoprecipitate (n = 14) and those who received fibrinogen concentrate (n = 20). The authors did not find any difference in blood loss, transfusion requirement, or need for a surgical hemostatic procedure. The first randomized controlled trial investigating the use of fibrinogen concentrate in PPH, in which 229 women participated, was published this year ^53^. The “FIB-PPH Trial”, as it is known, is a Danish multicenter placebo-controlled, double-blinded clinical trial evaluating whether initial treatment with fibrinogen concentrate (2 g) reduces the need for allogenic blood transfusion in PPH. No difference was observed between the two groups in RBC transfusion requirement up to 6 weeks postpartum or in any of the secondary predefined outcomes (total blood loss, total amount of blood transfused, occurrence or rebleeding, low hemoglobin level, a composite outcome of severe PPH, and RBC transfusion within 4 hours, 24 hours, and 7 days). However, women included in this trial had no acquired hypofibrinogenemia. Consequently, this study could draw conclusions only on the inefficacy of the use of fibrinogen concentrates as a pre-emptive treatment for severe PPH in patients with normofibrinogenemia.

Finally, the literature on the use of fibrinogen concentrate in non-obstetric hemorrhage only moderately suggests an efficacy on transfusion requirement and morbidity without formally proving it. Even if it appears to be a promising therapeutic, there is still no strong evidence that the use of fibrinogen concentrate would improve maternal outcomes in severe PPH. Moreover, the risk of thromboembolic events associated with the use of fibrinogen concentrate has never been explored in this context. Therefore, we still need valid data before administration of fibrinogen concentrate as a curative treatment of PPH can be firmly recommended.

## Additional strategies

Recombinant human FVIIa generated great hope several years ago when early case reports suggested immediate efficacy in refractory PPH ^54^. Unfortunately, randomized trials in trauma and more recently in obstetrics have shown only a moderate decrease in blood product consumption but no survival benefit, whereas the risk of thrombotic events seems to increase significantly ^55^.

Prothrombin complex concentrates contain several important coagulation factors and it has been suggested that they could replace FFP. This has been shown mainly in case reports or series in which coagulation factor deficit was detected by using POC viscoelastic tests in trauma ^47^ or traditional hemostatic tests in obstetric patients ^56^.

## Viscoelastic point-of-care coagulation monitoring

In current strategies, drugs and blood products are administered very early and with aggressive protocols. These are mostly “blind” techniques, since drugs or blood products are given either prophylactically or in response to severe blood loss but very often before the return of laboratory results can inform the physician of coagulation abnormalities. Moreover, blood product and, especially, FFP administration is aimed at globally correcting coagulation without a precise target.

However, it has been shown in recent years that it is possible to obtain coagulation tests results very early by using POC devices which are based mainly on viscoelastic techniques: thromboelastography (TEG) or thromboelastometry (ROTEM). These biological techniques can provide results within minutes and precisely inform the physician of the main hemostatic abnormalities. Physicians can thus direct treatment against precise targets and avoid (or reduce) the use of blood products. As shown above, hyperfibrinolysis and decreased fibrinogen concentration are important coagulation defects in massive hemorrhage and especially in obstetrics ^27^ and can be detected early by using viscoelastic techniques ^57^. Thus, early administration of fibrinogen concentrates can be guided by POC results rather than by the theoretical premise that these defects are very frequent. In a before-and-after study, Mallaiah *et al*. recently showed a significant decrease in blood product component use and a reduced incidence of circulatory overload with ROTEM-guided fibrinogen concentrate administration in major obstetric hemorrhage as compared with traditional care ^58^. There is currently no randomized study in obstetrics showing that this strategy decreases blood product use and improves outcomes, but small randomized studies performed in cardiovascular surgery have described positive results ^39, 41^. Increased use of these viscoelastic tests is likely to occur soon because although these tests remain relatively costly and there is a need for quality control of these machines, new versions, which are as easy to use as POC hemoglobin measurement tests, have been recently released.

## Teamwork and safety issues

PPH is a typical obstetric emergency situation that can develop rapidly and unexpectedly. Health-care professionals taking care of obstetric emergencies act as a team in which each provider uses his or her specific competencies. Moreover, because each obstetric emergency situation is a relatively rare event, even in high-level reference centers, providers have relatively few opportunities to train by self-experience and to evaluate and discuss how previous cases have been managed. During any emergency situation, communication and organizing the process of care are difficult tasks. It has been recognized that in many cases there is no clear leadership ^59^, and poor teamwork has been recognized as a major cause of poor outcome ^60^. For each team member, non-technical skills thus represent an important component of competency. Flin and Maran have described non-technical skills as two cognitive competencies (situational awareness and decision making) and two social competencies (teamwork and leadership) ^61^. Most of these competencies are universal and should be practiced by every health-care provider whatever his or her profession or grade. Unfortunately, using these four skills is difficult and does not come naturally to most humans. Recognition of these deficiencies and subsequent training are thus essential. Unfortunately, traditional teaching is almost ineffective to improve patients’ outcomes. Recently developed strategies have emerged to facilitate adoption of these non-technical skills in clinical practice. Interprofessional education is a relatively recent concept and is said to occur ‘when two or more professions learn with, from and about each other to improve collaboration and the quality of care’ ^62^. Interprofessional education is believed to be important for undergraduate students ^63^ but also for professionals working in clinical units to develop or maintain interprofessional collaboration. Team training is gaining popularity, as it is now recognized to improve quality of care ^64^. Team training as well as interprofessional education for students can be done through formal courses and meetings, but many studies have shown that simulation is effective to improve communication, teamwork, and patients’ outcomes ^65, 66^. Quality of care can also be improved by writing protocols that are made available to all providers ^67^ and through retrospective audits, which reduce the incidence of severe PPH and improve the application of recommendations ^68^.

## Conclusions

Until now, no study has proven that a specific transfusion strategy or the use of any pro-hemostatic agent would improve maternal outcomes in the context of PPH. Levels of evidence of TA and fibrinogen concentrate efficacy and safety in PPH are low. Randomized controlled trials in the context of severe PPH are difficult to perform, but there is room for studies of good quality to explore these therapeutic options.

## Abbreviations

FFP, fresh frozen plasma; POC, point-of-care; PPH, postpartum hemorrhage; RBC, red blood cell; TA, tranexamic acid.

## References

[1] Hogan MC Foreman KJ Naghavi M: Maternal mortality for 181 countries, 1980–2008: a systematic analysis of progress towards Millennium Development Goal 5. *Lancet.* 2010;375(9726):1609–23. 10.1016/S0140-6736(10)60518-1 20382417

[2] Knight M Kenyon S Brocklehurst P: Saving Lives, Improving Mothers' Care - Lessons learned to inform future maternity care from the UK and Ireland Confidential Enquiries into Maternal Deaths and Morbidity 2009–12.Oxford: University of Oxford,2014 Reference Source

[3] Enquète Nationale Confidentielle sur les Morts Maternelles, France 2007–2009. *Rapport du Comité National d'experts sur la Mortalité Maternelle (CNEMM)* 2013 Reference Source 10.1016/j.gofs.2024.02.005 38382838

[4] Zhang W Alexander S Bouvier-Colle M: Incidence of severe pre-eclampsia, postpartum haemorrhage and sepsis as a surrogate marker for severe maternal morbidity in a European population-based study: the MOMS-B survey. *BJOG.* 2005;112(1):89–96. 10.1111/j.1471-0528.2004.00303.x 15663404

[5] Belghiti J Kayem G Dupont C: Oxytocin during labour and risk of severe postpartum haemorrhage: a population-based, cohort-nested case-control study. *BMJ Open.* 2011;1(2):e000514. 10.1136/bmjopen-2011-000514 22189353 PMC3334825

[6] Borgman MA Spinella PC Perkins JG: The ratio of blood products transfused affects mortality in patients receiving massive transfusions at a combat support hospital. *J Trauma.* 2007;63(4):805–13. 10.1097/TA.0b013e3181271ba3 18090009

[7] Holcomb JB Jenkins D Rhee P: Damage control resuscitation: directly addressing the early coagulopathy of trauma. *J Trauma.* 2007;62(2):307–10. 10.1097/TA.0b013e3180324124 17297317

[8] Maegele M Lefering R Paffrath T: Changes in transfusion practice in multiple injury between 1993 and 2006: a retrospective analysis on 5389 patients from the German Trauma Registry. *Transfus Med.* 2009;19(3):117–24. 10.1111/j.1365-3148.2009.00920.x 19566668

[9] Borgman MA Spinella PC Holcomb JB: The effect of FFP:RBC ratio on morbidity and mortality in trauma patients based on transfusion prediction score. *Vox Sang.* 2011;101(1):44–54. 10.1111/j.1423-0410.2011.01466.x 21438884 PMC3155292

[10] Ho AM Dion PW Yeung JH: Prevalence of survivor bias in observational studies on fresh frozen plasma: erythrocyte ratios in trauma requiring massive transfusion. *Anesthesiology.* 2012;116(3):716–28. 10.1097/ALN.0b013e318245c47b 22270506

[11] Holcomb JB del Junco DJ Fox EE: The prospective, observational, multicenter, major trauma transfusion (PROMMTT) study: comparative effectiveness of a time-varying treatment with competing risks. *JAMA Surg.* 2013;148(2):127–36. 10.1001/2013.jamasurg.387 23560283 PMC3740072

[12] Holcomb JB Tilley BC Baraniuk S: Transfusion of plasma, platelets, and red blood cells in a 1:1:1 vs a 1:1:2 ratio and mortality in patients with severe trauma: the PROPPR randomized clinical trial. *JAMA.* 2015;313(5):471–82. 10.1001/jama.2015.12 25647203 PMC4374744

[13] Watson GA Sperry JL Rosengart MR: Fresh frozen plasma is independently associated with a higher risk of multiple organ failure and acute respiratory distress syndrome. *J Trauma.* 2009;67(2):221–7; discussion 228–30. 10.1097/TA.0b013e3181ad5957 19667872

[14] Spahn DR Bouillon B Cerny V: Management of bleeding and coagulopathy following major trauma: an updated European guideline. *Crit Care.* 2013;17(2):R76. 10.1186/cc12685 23601765 PMC4056078

[15] Saule I Hawkins N: Transfusion practice in major obstetric haemorrhage: lessons from trauma. *Int J Obstet Anesth.* 2012;21(1):79–83. 10.1016/j.ijoa.2011.09.009 22119633

[16] Alexander JM Sarode R McIntire DD: Whole blood in the management of hypovolemia due to obstetric hemorrhage. *Obstet Gynecol.* 2009;113(6):1320–6. 10.1097/AOG.0b013e3181a4b390 19461429

[17] Burtelow M Riley E Druzin M: How we treat: management of life-threatening primary postpartum hemorrhage with a standardized massive transfusion protocol. *Transfusion.* 2007;47(9):1564–72. 10.1111/j.1537-2995.2007.01404.x 17725718

[18] Skupski DW Lowenwirt IP Weinbaum FI: Improving hospital systems for the care of women with major obstetric hemorrhage. *Obstet Gynecol.* 2006;107(5):977–83. 10.1097/01.AOG.0000215561.68257.c5 16648399

[19] Butwick AJ Goodnough LT: Transfusion and coagulation management in major obstetric hemorrhage. *Curr Opin Anaesthesiol.* 2015;28(3):275–84. 10.1097/ACO.0000000000000180 25812005 PMC4567035

[20] Matteson KA Rahn DD Wheeler TL: Nonsurgical management of heavy menstrual bleeding: a systematic review. *Obstet Gynecol.* 2013;121(3):632–43. 10.1097/AOG.0b013e3182839e0e 23635628 PMC4414119

[21] Shakur H Roberts I Bautista R: Effects of tranexamic acid on death, vascular occlusive events, and blood transfusion in trauma patients with significant haemorrhage (CRASH-2): a randomised, placebo-controlled trial. *Lancet.* 2010;376(9734):23–32. 10.1016/S0140-6736(10)60835-5 20554319

[22] Ker K Edwards P Perel P: Effect of tranexamic acid on surgical bleeding: systematic review and cumulative meta-analysis. *BMJ.* 2012;344:e3054. 10.1136/bmj.e3054 22611164 PMC3356857

[23] Novikova N Hofmeyr GJ Cluver C: Tranexamic acid for preventing postpartum haemorrhage. *Cochrane Database Syst Rev.* 2015; (6): CD007872. 10.1002/14651858.CD007872.pub3 26079202 PMC11236425

[24] Ducloy-Bouthors AS Jude B Duhamel A: High-dose tranexamic acid reduces blood loss in postpartum haemorrhage. *Crit Care.* 2011;15(2):R117. 10.1186/cc10143 21496253 PMC3219400

[25] WHO Guidelines Approved by the Guidelines Review Committee: WHO recommendations for the prevention and treatment of postpartum haemorrhage.Geneva, Switzerland,2012. 23586122

[26] Shakur H Elbourne D Gülmezoglu M: The WOMAN Trial (World Maternal Antifibrinolytic Trial): tranexamic acid for the treatment of postpartum haemorrhage: an international randomised, double blind placebo controlled trial. *Trials.* 2010;11:40. 10.1186/1745-6215-11-40 20398351 PMC2864262

[27] de Lloyd L Bovington R Kaye A: Standard haemostatic tests following major obstetric haemorrhage. *Int J Obstet Anesth.* 2011;20(2):135–41. 10.1016/j.ijoa.2010.12.002 21439811

[28] Charbit B Mandelbrot L Samain E: The decrease of fibrinogen is an early predictor of the severity of postpartum hemorrhage. *J Thromb Haemost.* 2007;5(2):266–73. 10.1111/j.1538-7836.2007.02297.x 17087729

[29] Cortet M Deneux-Tharaux C Dupont C: Association between fibrinogen level and severity of postpartum haemorrhage: secondary analysis of a prospective trial. *Br J Anaesth.* 2012;108(6):984–9. 10.1093/bja/aes096 22490316

[30] Collis RE Collins PW: Haemostatic management of obstetric haemorrhage. *Anaesthesia.* 2015;70(Suppl 1):78–86,e27–8. 10.1111/anae.12913 25440400

[31] Bornikova L Peyvandi F Allen G: Fibrinogen replacement therapy for congenital fibrinogen deficiency. *J Thromb Haemost.* 2011;9(9):1687–704. 10.1111/j.1538-7836.2011.04424.x 21711446

[32] Fenger-Eriksen C Anker-Møller E Heslop J: Thrombelastographic whole blood clot formation after *ex vivo* addition of plasma substitutes: improvements of the induced coagulopathy with fibrinogen concentrate. *Br J Anaesth.* 2005;94(3):324–9. 10.1093/bja/aei052 15608046

[33] Fries D Krismer A Klingler A: Effect of fibrinogen on reversal of dilutional coagulopathy: a porcine model. *Br J Anaesth.* 2005;95(2):172–7. 10.1093/bja/aei160 15923269

[34] Cui Y Hei F Long C: Perioperative monitoring of thromboelastograph on blood protection and recovery for severely cyanotic patients undergoing complex cardiac surgery. *Artif Organs.* 2010;34(11):955–60. 10.1111/j.1525-1594.2010.01148.x 21092037

[35] Fenger-Eriksen C Jensen TM Kristensen BS: Fibrinogen substitution improves whole blood clot firmness after dilution with hydroxyethyl starch in bleeding patients undergoing radical cystectomy: a randomized, placebo-controlled clinical trial. *J Thromb Haemost.* 2009;7(5):795–802. 10.1111/j.1538-7836.2009.03331.x 19320829

[36] Galas FR de Almeida JP Fukushima JT: Hemostatic effects of fibrinogen concentrate compared with cryoprecipitate in children after cardiac surgery: a randomized pilot trial. *J Thorac Cardiovasc Surg.* 2014;148(4):1647–55. 10.1016/j.jtcvs.2014.04.029 24951020

[37] Karlsson M Ternström L Hyllner M: Prophylactic fibrinogen infusion reduces bleeding after coronary artery bypass surgery. A prospective randomised pilot study. *Thromb Haemost.* 2009;102(1):137–44. 10.1160/TH08-09-0587 19572078

[38] Lancé MD Ninivaggi M Schols SE: Perioperative dilutional coagulopathy treated with fresh frozen plasma and fibrinogen concentrate: a prospective randomized intervention trial. *Vox Sang.* 2012;103(1):25–34. 10.1111/j.1423-0410.2011.01575.x 22211833

[39] Rahe-Meyer N Solomon C Hanke A: Effects of fibrinogen concentrate as first-line therapy during major aortic replacement surgery: a randomized, placebo-controlled trial. *Anesthesiology.* 2013;118(1):40–50. 10.1097/ALN.0b013e3182715d4d 23249928

[40] Tanaka KA Egan K Szlam F: Transfusion and hematologic variables after fibrinogen or platelet transfusion in valve replacement surgery: preliminary data of purified lyophilized human fibrinogen concentrate versus conventional transfusion. *Transfusion.* 2014;54(1):109–18. 10.1111/trf.12248 23718572

[41] Ranucci M Baryshnikova E Crapelli GB: Randomized, double-blinded, placebo-controlled trial of fibrinogen concentrate supplementation after complex cardiac surgery. *J Am Heart Assoc.* 2015;4(6):e002066. 10.1161/JAHA.115.002066 26037084 PMC4599543

[42] Aubron C Reade MC Fraser JF: Efficacy and safety of fibrinogen concentrate in trauma patients--a systematic review. *J Crit Care.* 2014;29(3):471.e11–7. 10.1016/j.jcrc.2013.12.011 24508201

[43] Fenger-Eriksen C Lindberg-Larsen M Christensen AQ: Fibrinogen concentrate substitution therapy in patients with massive haemorrhage and low plasma fibrinogen concentrations. *Br J Anaesth.* 2008;101(6):769–73. 10.1093/bja/aen270 18818192

[44] Innerhofer P Westermann I Tauber H: The exclusive use of coagulation factor concentrates enables reversal of coagulopathy and decreases transfusion rates in patients with major blunt trauma. *Injury.* 2013;44(2):209–16. 10.1016/j.injury.2012.08.047 23000050

[45] Nienaber U Innerhofer P Westermann I: The impact of fresh frozen plasma vs coagulation factor concentrates on morbidity and mortality in trauma-associated haemorrhage and massive transfusion. *Injury.* 2011;42(7):697–701. 10.1016/j.injury.2010.12.015 21392760

[46] Schöchl H Nienaber U Maegele M: Transfusion in trauma: thromboelastometry-guided coagulation factor concentrate-based therapy versus standard fresh frozen plasma-based therapy. *Crit Care.* 2011;15(2):R83. 10.1186/cc10078 21375741 PMC3219338

[47] Schöchl H Nienaber U Hofer G: Goal-directed coagulation management of major trauma patients using thromboelastometry (ROTEM)-guided administration of fibrinogen concentrate and prothrombin complex concentrate. *Crit Care.* 2010;14(2):R55. 10.1186/cc8948 20374650 PMC2887173

[48] Guasch E Alsina E Díez J: Hemorragia obstétrica: estudio observacional sobre 21.726 partos en 28 meses. *Rev Esp Anestesiol Reanim.* 2009;56(3):139–46. 10.1016/S0034-9356(09)70356-1 19408780

[49] Weinkove R Rangarajan S: Fibrinogen concentrate for acquired hypofibrinogenaemic states. *Transfus Med.* 2008;18(3):151–7. 10.1111/j.1365-3148.2008.00854.x 18598277

[50] Weiss G Lison S Glaser M: Observational study of fibrinogen concentrate in massive hemorrhage: evaluation of a multicenter register. *Blood Coagul Fibrinolysis.* 2011;22(8):727–34. 10.1097/MBC.0b013e32834cb343 22024795

[51] Kikuchi M Itakura A Miki A: Fibrinogen concentrate substitution therapy for obstetric hemorrhage complicated by coagulopathy. *J Obstet Gynaecol Res.* 2013;39(4):770–6. 10.1111/j.1447-0756.2012.02058.x 23278972

[52] Ahmed S Harrity C Johnson S: The efficacy of fibrinogen concentrate compared with cryoprecipitate in major obstetric haemorrhage--an observational study. *Transfus Med.* 2012;22(5):344–9. 10.1111/j.1365-3148.2012.01178.x 22994449

[53] Wikkelsø AJ Edwards HM Afshari A: Pre-emptive treatment with fibrinogen concentrate for postpartum haemorrhage: randomized controlled trial. *Br J Anaesth.* 2015;114(4):623–33. 10.1093/bja/aeu444 25586727

[54] Bouwmeester FW Jonkhoff AR Verheijen RH: Successful treatment of life-threatening postpartum hemorrhage with recombinant activated factor VII. *Obstet Gynecol.* 2003;101(6):1174–6. 10.1016/S0029-7844(03)00350-8 12798521

[55] Lavigne-Lissalde G Aya AG Mercier FJ: Recombinant human FVIIa for reducing the need for invasive second-line therapies in severe refractory postpartum hemorrhage: a multicenter, randomized, open controlled trial. *J Thromb Haemost.* 2015;13(4):520–9. 10.1111/jth.12844 25594352

[56] Glynn JC Plaat F: Prothrombin complex for massive obstetric haemorrhage. *Anaesthesia.* 2007;62(2):202–3. 10.1111/j.1365-2044.2007.04972.x 17223835

[57] Huissoud C Carrabin N Audibert F: Bedside assessment of fibrinogen level in postpartum haemorrhage by thrombelastometry. *BJOG.* 2009;116(8):1097–102. 10.1111/j.1471-0528.2009.02187.x 19459866

[58] Mallaiah S Barclay P Harrod I: Introduction of an algorithm for ROTEM-guided fibrinogen concentrate administration in major obstetric haemorrhage. *Anaesthesia.* 2015;70(2):166–75. 10.1111/anae.12859 25289791

[59] Guise JM Segel SY Larison K: STORC safety initiative: a multicentre survey on preparedness & confidence in obstetric emergencies. *Qual Saf Health Care.* 2010;19(6):e41. 10.1136/qshc.2008.030890 21127088

[60] Mazzocco K Petitti DB Fong KT: Surgical team behaviors and patient outcomes. *Am J Surg.* 2009;197(5):678–85. 10.1016/j.amjsurg.2008.03.002 18789425

[61] Flin R Maran N: Basic concepts for crew resource management and non-technical skills. *Best Pract Res Clin Anaesthesiol.* 2015;29(1):27–39. 10.1016/j.bpa.2015.02.002 25902464

[62] (CAIPE) CftAoIE.2016 Reference Source

[63] Lapkin S Levett-Jones T Gilligan C: A systematic review of the effectiveness of interprofessional education in health professional programs. *Nurse Educ Today.* 2013;33(2):90–102. 10.1016/j.nedt.2011.11.006 22196075

[64] Weaver SJ Dy SM Rosen MA: Team-training in healthcare: a narrative synthesis of the literature. *BMJ Qual Saf.* 2014;23(5):359–72. 10.1136/bmjqs-2013-001848 24501181 PMC3995248

[65] Fung L Boet S Bould MD: Impact of crisis resource management simulation-based training for interprofessional and interdisciplinary teams: A systematic review. *J Interprof Care.* 2015;29(5):433–44. 10.3109/13561820.2015.1017555 25973615

[66] Phipps MG Lindquist DG McConaughey E: Outcomes from a labor and delivery team training program with simulation component. *Am J Obstet Gynecol.* 2012;206(1):3–9. 10.1016/j.ajog.2011.06.046 21840493

[67] Shields LE Smalarz K Reffigee L: Comprehensive maternal hemorrhage protocols improve patient safety and reduce utilization of blood products. *Am J Obstet Gynecol.* 2011;205(4):368.e1–8. 10.1016/j.ajog.2011.06.084 22083059

[68] Dupont C Deneux-Tharaux C Touzet S: Clinical audit: a useful tool for reducing severe postpartum haemorrhages? *Int J Qual Health Care.* 2011;23(5):583–9. 10.1093/intqhc/mzr042 21733978 PMC3428268

